# Spinal Anæsthesia
*A paper read at a meeting of the Society on Wednesday, 13th January, 1937.


**Published:** 1937

**Authors:** A. W. Adams

**Affiliations:** Surgeon to the Bristol Royal Infirmary


### SPINAL ANÆSTHESIA*

BY

A. W. Adams, F.R.C.S.,

*Surgeon to the Bristol Royal Infirmary.*

---

The value and importance of spinal anæsthesia is now well established. The object of this communication is to discuss the indications and limitations of this procedure. The few special requisites I described in detail in the *British Medical Journal* in 1931.[1] I have, however, been led to radical alterations in dosage and in method of distributing the anæsthetic, spinocain.

The aseptic technique is followed fully as in ordinary operations. Meningitis has not occurred in this series, but there have been a few cases of severe headache. These may last for a week, and one had mild pyrexia. I suppose this is a simple inflammatory response. Failure to tap the theca with the patient of whatever age in the lateral position is almost unknown. Undeviating adherence to the exact mid-line is the path to sure and swift puncture. Usually the point of the needle needs a few degrees' tilt in a cephalic direction. 0·5 c.cm. of spinocain is used between the third and fourth lumbar spines for the peno-scrotal and coccygeal areas, and the table immediately sloped to 20° Trendelenberg position,

* A paper read at a meeting of the Society on Wednesday, 13th January, 1937.

the patient being turned on to his back during the two minutes' induction. For operations up to and including the knee 1 c.cm. between the second and third lumbar spines is used. As hernial operations may involve dragging on the parieto-visceral peritoneum a full abdominal anæsthesia is probably best, as for other forms of laparotomy. The space between the first and second lumbar spines is chosen, the table inclined in the reverse Trendelenberg position until the *spine* makes an angle of 5° with the horizontal and 1·35 c.cm. slowly injected. It will rightly be inferred that admixture of cerebro-spinal fluid is not essential for successful anæsthesia; but an equal part of it is desirable. It warms the cold spinocain and prevents a strong solution reaching nerves at the site of injection. Induction takes about four minutes, but in rare cases may take fifteen to twenty-five minutes. After the lapse of a minute the table is levelled, while at two minutes a head-down slope of 5° to 10° is assumed, the patient gently rolled over on to the back and one pillow permitted for the head. Renal cases only differ in remaining in the lateral position for the operation. 1·3 c.cm. is the usual dose of spinocain, but up to 1·4 c.cm. is sometimes given according to the level of operation and type of patient. Though fluid often drips from the needle after puncture, it sometimes happens that none can be drawn into the syringe. In such cases the syringe is detached, and if confirmatory drops still flow, injection may be made with confidence. In about one per cent. of patients anæsthesia is perfect for the parietal incision but not for the subjacent viscera. This disappointing fact is due presumably to sensory function in the vago-sympathetic. Novocain infiltration of the mesentery makes good the defect in some cases, and it is a useful routine adjunct in the upper abdomen. In a further one per cent. even somatic anæsthesia fails above the lower abdomen. This rare deficiency is the price paid for a "safety-first" policy and insurance against undue ascent of anæsthesia. Experience in the use of this drug leads to the conclusion that nerves in different patients vary in grade between gross examples of resistance to novocain on the one hand to marked susceptibility on the other. To this factor rather than to errors in technique must be attributed occasional variation in results in skilful hands. A comparable phenomenon of the central nervous system is met with in the action of ether or chloroform, to which some patients yield but stubbornly, and indeed may never resign themselves properly during the whole of a major operation.

The duration of anæsthesia averages sixty to seventy minutes. I once noticed returning sensibility at thirty-five minutes in a case of cholecystectomy, but on many occasions anæsthesia has persisted for two hours or longer.

The patient is returned to bed with the foot raised on six-inch blocks for an hour, and special watch is kept where, owing to a short operation, the blood-pressure is not yet normal.

The anæsthetic agent is injected at one spot in the theca. Its spread thence depends on diffusion and that, in turn, partly on dosage. Other factors are at work which complicate its distribution. First, nerve tissue has an absorptive action on novocain and fixes it. Secondly, the gliadin included in the compound called spinocain retards the process of diffusion. Finally, alcohol (14·5 per cent.) in the solution renders it lighter than cerebrospinal fluid, but this influence is inconstant in degree because the density of the *latter*

is slightly variable. However, cerebrospinal fluid is always sufficiently heavier for the initial trend of the injection to be upwards on entering a sloping theca. This is the rationale for the foregoing instructions as to the inclination of the spine during administration and the varied level of interspinous spaces chosen for puncture. When the drug ascends beyond a certain level the anæsthesia is good but danger is nigh. This was my early experience, and though no deaths occurred, was daunting enough to deter further efforts, but the improved method subsequently introduced by Pitkin inspired me to renewed trial.

### CASES OPERATED ON UNDER SPINAL ANÆSTHESIA (SPINOCAIN).

STATISTICAL SUMMARY TO 30TH JUNE, 1936.

| | |
|---|---|
| UPPER ABDOMEN : | |
| Stomach, duodenum, jejunum .. .. .. | 87 |
| (of which 13 were partial gastrectomies) | |
| Gall-bladder .. .. .. .. .. | 60 |
| Spleen .. .. .. .. .. .. | 3 |
| Kidney and ureter .. .. .. .. | 48 |
| LOWER ABDOMEN : | |
| Colon .. .. .. .. .. .. | 90 |
| Abdomino-perineal excision of rectum .. | 7 |
| Acute intestinal obstruction (excluding hernia) | 43 |
| Appendix .. .. .. .. .. | 170 |
| Hernia (including Gallie's operation and strangulated hernia) .. .. .. .. | 133 |
| Laparotomy .. .. .. .. .. | 67 |
| External genitalia .. .. .. .. | 37 |
| Bladder .. .. .. .. .. .. | 31 |
| Prostatectomy .. .. .. .. .. | 136 |
| SACRAL AREA : | |
| Perineal excision of rectum .. .. .. | 7 |
| Anal, peri-anal, and coccygectomy .. .. | 58 |
| Cystoscopic procedures .. .. .. | 360 |

| | |
|---|---|
| LOWER LIMB : | |
| (Including fractures, dislocations, deranged knees, osteomyelitis, etc.) .. .. .. | 192 |
| TOTAL (of which 48 were children under 12 years) | 1,529 |

### DANGERS AND DRAWBACKS.

*Dangers.*—The chief is shock. This is partly due to the nervous susceptibility of the subject, but is mainly due to lowered blood-pressure. Its degree is proportional to the extent of paralysis of those fibres in the spinal nerves destined for the sympathetic system and so for vaso-constrictor muscles. The rise of temperature in the legs is the obvious result of this flushing. These nerve fibres derive from the spinal segments from the second dorsal to the second lumbar. As abdominal innervation extends as high as the sixth dorsal segment, the drug must reach at least that level, and for complete confidence a few segments higher. In actual practice anæsthesia usually ends about the nipple level. I have not known miosis nor palsy in the hands using the doses here advocated, and it is rare to hear patients complain of any paræsthesia in the fingers. Not only does restricting the spread of the drug beyond the dorsal region guard against excessive sympathetic palsy, but it protects respiratory movements by preserving intact the functions of the phrenic nerve.

The ideal is to steer a middle course between falling blood-pressure and inadequate anæsthesia. Of the two obviously the former is to be avoided at all costs. Hence the dose of spinocain has been lowered till the minimum necessary for achieving anæsthesia was reached. So near the minimum is it that in a few per cent. it is inadequate. In compensation for this the method insures against fatalities. Even so, any threats of failing pulse must be carefully looked for as an abdominal operation proceeds, and the following precautions should be taken :—

PRECAUTIONS AGAINST SHOCK.

1. Record the blood-pressure prior to the operation and avoid spinal anæsthesia if it is sub-normal, e.g. with internal hæmorrhage of ruptured spleen, liver or ectopic gestation. Cogent arguments in its favour would be needed before using the method in patients with a systolic pressure below 110 mm. Hg. In the case of children reliance is placed on the resilience of the circulatory mechanism, and the blood-pressure is not tested.

2. Premedication with pressor substances, such as ephedrine, is a routine procedure.

3. Repeated vomiting during operation, exhaustion and air hunger are to be regarded as possible indications of serious fall in systolic pressure. They are of less significance in younger patients. Have in readiness an emergency syringe for intravenous injection containing 1 c.cm. of 1 in 1,000 adrenalin. This is given if the pulse is nearing imperceptibility at the wrist. The surgeon may finger the ilio-femoral artery. About a minute after adrenalin enters the vein the most dramatic invigoration of the circulation ensues. The pulse bounds, colour replaces pallor, and the wound bleeds. The operation is then completed. On one occasion this procedure failed, but the pulse returned strongly after intracardiac injection of adrenalin combined with massage of the heart through the diaphragm. Adrenalin is most magical among medicines. For milder degrees of prostration a subcutaneous dose suffices. Obviously a full Trendelenberg position and artificial respiration may be useful adjuncts.

4. Before the incision, in a case where spinal anæsthesia is strongly indicated but liability to undue shock is suspected, the institution of continuous intravenous saline would be an added safeguard.

5. Restrict the use of narcotics and general anæsthetics. When a serious degree of shock and lowered vitality is present the aspect of the conscious patient undergoes obvious changes. This valuable warning gives the opportunity for deliberate use of the restorative measures already referred to and kept in readiness. On the other hand, when spinal is combined with any other form of general anæsthetic narcosis is apt to mask the developing stages of such deterioration. The signals of distress are absent, until with alarming suddenness a profoundly grave state declares itself and demands instant resuscitatory measures. Such occasions, fortunately, are rare but memorable. Therefore, where we are working with frail subjects it is wise to keep the anæsthesia as simple as possible, and only employ additional "general" if the spinal proves inadequate.

Since the case (carcinoma coli) reported in 1931[2] to the end of the period covered by these statistics I have had no fatality, save once where spinal and nitrous oxide anæsthesia were combined. The autopsy on this man of 63 showed extreme flabbiness of the heart muscle and gross dilatation of the auriculo-ventricular rings. The many features that go to compose the circulatory picture make it complex. There is a wide range of blood-pressure, e.g. a successful prostatectomy in a plethoric man of 70 years who had blood-pressure of 240 mm. Hg., the varying resilience of the vessels and the subtle changes in the myocardium. An infallible pre-operative assessment of cardio-vascular functions is still a remote ideal, and the only way to escape fatality from this source is to err on the safe side in regard to dosage.

Novocain sensitivity and the reverse obviously play an important part. A slightly sub-average dose might create a profound effect on the circulation in the susceptible subject, as well as giving full anæsthesia. Per contra, in the resistant even the average dose might barely cover the requisite area and would scarcely make an impression on the pulse.

By observing the spread of anæsthesia in the conscious after spinal injection and adjusting the tilt of the table accordingly, one might hope that the spread of the drug would be controllable in accordance with the particular response of each individual. Unfortunately the intrathecal diffusion and fixation is too rapid.

In further proof of the assertion that the doses recommended are minimal is the fact that occasionally during laparatomy the legs of the patient move.

A young man lay contented, when I extended the incision for radical orchidectomy to the rib margin. Suddenly, as the cord was lifted near the internal inguinal ring, he felt two or three flashes of pain in the rectum. This was during traction on the unparalysed prerectal end of the vas deferens. Such phenomena are explained by the fact that the bulk of the injection is so small that it does not reach the lowest spinal nerves.

It is evident that some control of caudal as well as cephalic spread is possible, concurrently with full anæsthesia of the intermediate spinal nerves. This "zonal" anæsthesia may be the ideal for the supra-pelvic operations. Of course, where the surgeon expects his manipulations to transgress the pelvis, even if only for a moment or two during exploration, the patient needs the safeguard of a few whiffs of general anæsthetic.

CONTRA-INDICATIONS.

Patients with sub-normal blood-pressure and the acutely exsanguinated are ill-fitted to cope with the added depression of dorsal spinal anæsthesia, while the chronic asthmatic or emphysematous chest has so reduced a respiratory exchange that I have proved it is dangerous to deprive such of intercostal movement, and local or general anæsthesia should be used. Obviously unfavourable is the poor surgical risk, like an old patient of mine, who died under spinal anæsthesia since the statistics for this paper were compiled. This is his story:

" E.R.D. in the summer of 1934, when 63, healed well from cholecystostomy for stones and subsequent choledochotomy for cure of biliary fistula. Spinal anæsthesia was successful for both. By 1936 he had four "strokes," and on 27th November, 1936, a third laparotomy for apparent acute biliary trouble. Spinal anæsthesia was used, but he died during the operation, which disclosed nothing beyond free fluid and adhesions. Autopsy gave the clue to this tragedy. In the left ventricle was an old infarct and in its cavity a large organized thrombus. Shrivelled infarcts scarred the spleen, and tough cirrhosis the liver, while the cortex of the kidneys had almost disappeared with interstitial nephritis."

In view of such findings the result might well have been the same under general anæsthesia.

Another type emerges if a study is made of those who have given anxiety on the table. Their chances of, at any rate immediate, survival are likely to be greater under an alternative to spinal anæsthesia. I mean the chronic invalid whose main alimentary or urinary passages have long been fouled by growths, especially fungating ones, or sepsis. Their reserves have been sapped, their powers of resilience are in abeyance, and they come to operation with heart and other tissues degenerated by toxæmia and secondary anæmia and with their metabolism torpid. If collapse or worse occur on the table after a skilfully administered spinal anæsthetic, then their victims will be among such subjects.

It behoves us to be convinced that the advantages justify the slightly added risk of spinal anæsthesia before employing it in this class of patient. Favourably contrasting with these is the acute abdominal case, whether arising in the healthy or complicating chronic illness, for the sudden assault on their organisms has roused their forces of resistance to full vigour. They are well able to contend with the transient depressive influence of the spinal anæsthetic. This favourable reactionary period is followed all too soon by rapid deterioration, and though still acutely ill, the patient is leaving the level of vigour where he can safely avail himself of the advantages offered by this method. For instance, once the acute intestinal obstruction case has passed the zenith of reaction he will need very careful piloting if he has a spinal anæsthetic. Often his needs will be better met by a local or skilfully administered general anæsthetic. Spinal anæsthesia usefully reminds us of the need for promptitude in dealing with the acute abdomen.

Again, sufferers from gall-stones, who oftener come to operation "in the cold," have fared well, as shown by preceding detailed statistics for two reasons :—

1. Periods of good nutrition intervene between their intermittent attacks.

2. The main alimentary thoroughfare is not (directly) involved.

Of the peptic ulcer class it is notable that younger patients figure largely, the disease exerts a baleful influence less continuously, and degenerative processes in the body are correspondingly less advanced. The subperitoneal site of operation shields the prostate patient from the indirect effects of surgical manipulations and he is often in fair general health.

*Drawbacks.*—In the foregoing is set forth how to avoid and to treat shock, the chief difficulty of spinal anæsthesia. Slighter drawbacks call for mention. Occasionally the patient complains of paræsthesia in the lower limbs, but this ceases after a few minutes. Later itching occurs in a few cases and may annoy the patient longer. What troubles the theatre staff more than the patient is the occasional evacuation of fæces on the table. It happens in a few cases, mainly emergencies with stagnant colons, and is due to paralysis of the sympathetic innervation of the bowel and the unopposed activity of the vagus. Where it is expected suitably placed pads will control the outflow.

*Headache* is uncommon, but as with other rare phenomena in medicine, two or three cases are apt to appear within a short period, separated by an interval so long that one forgets the existence of it. In the hope of minimizing this trouble a strict maintenance of the recumbent position is enjoined during the day following anæsthesia, but the effects of posture in this regard are questionable. Complaint of headache is almost unheard of from out-patient cystoscopy cases. Yet after a few hours' recumbency they leave hospital as ambulatory cases. Palsy of the sixth cranial nerve lasted for three months in a case reported in my first series. No other paralysis has occurred save temporary, almost total, deafness. This was in a man, H.G., aged 53 years, who on 5th October, 1935, came to operation desperately ill with late intestinal obstruction due to a "band." After several weeks hearing returned.

Backache due to trauma to the inter-spinous ligaments from the needle is rare with skilful puncture and passes off.

### THE DEBATABLE TERRITORY OF THE UPPER ABDOMEN.

From what I have written it may rightly be inferred that in the lower abdomen spinal anæsthesia is sound practice. In the area presided over by the lumbo-sacral nerves it surpasses any other method for routine purposes. In the upper abdomen we reach a place of dissension. The peculiar difficulties in the upper abdomen are the unmitigated functions of the vagus and phrenic nerves, which are apt to make the patient grizzle and cause the surgeon concern. Inevitably a little pulling occurs on the stomach and liver, which is liable to provoke resentment or nausea, while movements transmitted through the diaphragm to thoracic contents may be distressing and make the patient feel ill. This hindrance is not insuperable, but the fact that the remedy is not a simple one partly explains why spinal anæsthesia is less popular for operations in the upper abdomen than in the lower. Time has to be given for deliberate judgment on each case, and one of several adjuvants invoked. It might be thought that what is wanted to silence the troubles of this unparalysed phrenico-vagal territory is to force the ascent of spinocain, either by increased slope of the spine or greater doses, so high as to catch these nerves at their origin. It would be sheer folly to treat the problem so, since with the spinal palsy reaching no higher than the fourth dorsal the maximum permissible fall of blood-pressure may ensue. To press the drug a little because of occasional failure of upper abdominal palsy is fallacious and might even be fatal.

My first anxiety was to make spinal anæsthesia safe, and having practised it without any complicating factor of pre- or inter-medication I am in a position to judge its share in regard to any deterioration of the patient's state during an operation. To such a safe dose it is helpful, though not always essential, to add a routine infiltration of 1 per cent. novocain into the lesser omentum. This will be carried well round the cardiac end of the stomach in gastric cases and soaks up to the portal fissure in gall-stone operations. The patient is thus largely protected from phrenico-vagal irritation, and in many cases may go through the operation without further medication. Often more is needed, especially in allaying the apprehensions of women; but the consideration of this is left to a subsequent stage, while here the advantages of spinal anæsthesia in this sphere are discussed.

### GASTRO-DUODENAL AND BILIARY SURGERY.

Sufferers from chronic gastro-duodenal ulcer or cancer of the stomach are apt to be toxic, pale and chronic invalids. The operations required for their relief are formidable. Any doubts of this are dispelled by the mortality rate recently published by Tidy.[3] That the general anæsthetic has much to do with the disasters is suggested by the various alternatives to it that have been tried of late. Among these are splanchnic and, most recently, wholly local anæsthesia as advocated by Finsterer. Spinal anæsthesia confers benefits on patients whose liver, kidney, cardiac or respiratory functions are at low ebb. The vital organs are spared the disadvantages of super-added intoxication,[4] and the surgeon gains complete relaxation, that is, maximum access with minimum trauma, and welcome stillness. It is rapidly administered, and avoids the sodden state of the parietal tissues which at times is a drawback of local infiltration. Before dismissing such an ally, therefore, the doctor wants to be sure of his reasons.

Need the surgeon forbid the patient the advantages of spinal anæsthesia in gastric operations because it is risky ? Among my own cases (from January, 1931, onwards), many of which were acute perforations and partial gastrectomies, there has been no fatality at operation. However, where local anæsthesia is applicable it may be more ideal, is safe, and gives similar ease of access ; but the more tedious nature of the method and the acutely inflamed intra-peritoneal state make local anæsthesia difficult for perforated ulcers.

The other main sphere of upper abdominal work is biliary surgery. In these, as in gastric cases, the surgeon is often called upon to perform a major operation on a toxic subject. The myocardial and respiratory functions are apt to be defective, while the peculiar dangers of hepatic deficiency and jaundiced tissues are frequent accompaniments. It is in such subjects that an operation renowned as dark, dangerous and difficult is wont to be needed. Naturally surgeons welcome a method which spares such patients the added strain which general anæsthesia throws on their impaired metabolism. Spinal anæsthesia affords this boon, and at the same time easier access to the bile-ducts and subdued respiratory movements when working in the depths. Usually local infiltration is needed around the ducts, and often inhalations of general anæsthetic help the nervous subject during deep manipulations. Here spinal anæsthesia converts strenuous toil into smooth achievement.

Two leading surgeons, desirous of improving on general anæsthesia, tried it for gall-stones. Both forsook it, they told me, because of deaths on the table. It is possible that these disasters were due to improper administration and were not inevitable sequelæ of spinal anæsthesia. I quote from my own figures, drawing attention to the average age of the patients.

TABLE II.

*Biliary Operations.*

| | Total Nos. | C.B. Duct Obstn. present in | | Average Age. | Deaths. | |
|---|---|---|---|---|---|---|
| To 30.6.36 | 62 | 11 | 17·7% | 56 | 5 | 8·1% |
| From 30.6.36 To 37 | 16 | 3 | 18·75% | 55·25 | 0 | 0% |
| | 78 | 14 | 18% | 55·8 | 5 | 6·4% |

Most were operated on with purely spinal anæsthesia, not because they were good surgical risks, but rather the reverse. Also, I have added to the table the cases subsequent to 30th June, 1936, with a welcome improvement in the mortality rate. In the original compilation there were 62 cases, and in 51 the operation included cholecystectomy and routine exploration of the common bile-duct. It seems fair to add a few notes on the type of patient and the cause of the five fatalities.

H.O., male, aged 71, was a poor risk, having but partially regained mental and physical health after a stroke three years previously. He progressed smoothly after removal of the gall-bladder for stones and exploration of the common bile-duct. At the end of two weeks pulmonary embolism occurred, and five days later a second attack of this killed him.

W.H., male, aged 90, was found to have a ruptured gall-bladder packed with stones. He made a good recovery for a week, had acute retention of urine, and slowly sank and died about the seventeenth day after operation.

F.A., female, aged 79, with torsion of gall-bladder, convalesced smoothly for three days, and died suddenly on the fourth day after operation from heart failure.

The other two deaths followed closely on operation. One, a male aged 57, died twenty-four hours after, and the other, a female aged 52, died two days later. The post-mortem examination in the latter showed fatty degeneration of the heart, liver and kidneys.

There is no reason to think these patients would have survived had general anæsthesia been used. On the other hand, I do think that the remaining fifty-five cases who recovered might have been less successful had they been denied the advantages of spinal anæsthesia.

It seems fair, therefore, to claim in upper abdominal work under spinal compared with general anæsthesia that (*a*) the dangers are no greater, (*b*) the chance of the patient's ultimate recovery is as great, (*c*) technical perfection is likelier and recurrent biliary troubles therefore fewer.

### THE PSYCHOLOGICAL ASPECT.

Here is another fundamental factor involved in this matter. Though considered last, it is of first importance. Many critics, medical and lay, express abhorrence of being conscious while undergoing operations. Although a natural, it is not necessarily a profound view. The patient and the surgeon need to realize the truth that the price paid for temporary oblivion is a considerably greater risk of ultimate failure, in support of which contention cogent arguments have already been given.

The practice of Finsterer, one of the great gastric surgeons, endorses it further, and shows that patients' scruples on the score of consciousness may be overcome. He employs merely local anæsthesia for partial gastrectomy. This proves that with a little persuasion it is possible to satisfy the patient's mental needs as well as the bodily, so that he feels that this method is not only more beneficial but also sufficiently agreeable. Emphatically the demands on the doctor are far greater than is usual with inhalation anæsthesia. In fact, nothing less than exceptional exercise of his imagination, and the use of every art and all the tact the anæsthetist commands, suffice for the needs of the anxious patient. With this ideal in view, a little experience would make easy what at first seems burdensome. A share in this responsibility rests on all in the theatre.

As a rule the higher the level of operation the livelier the patient's awareness. Therefore, although this factor is relatively insignificant in the lumbo-sacral levels, it may be important in the hypogastric, and often is a pressing need when the surgeon is working in the upper abdomen. The encouraging words and hand-grasp of the anæsthetist during the ordeal of operation give the patient the comforting reassurance such as a hand-rail affords to one crossing a foot-bridge. Obviously a lumbo-sacral anæsthetic like a broad bridge means little risk, but where consciousness of the serious nature of the proceedings deepens, as in major abdominal operations, it is as if the plank is narrow. Then to feel that hand-rail is a matter of vital importance to nervous patients.

As the problem arises most acutely in these, its discussion has been postponed to this point. It is solved by grouping patients according to their mental make-up and the nature of their operation.

*A.*—Where an alternative to general anæsthesia is almost indispensable.

When patients are told that for the best result it is virtually essential for them to give their conscious collaboration at the operation almost all consent to spinal anæsthesia. Thus, when the breath is held during ascending pyelography a crisp picture is possible as diaphragmatic movements are eliminated. Where plasters have been applied to the pelvis and legs the patient who can look after the upper part of his body may facilitate the surgeon's work. The conscious patient requires less nursing attention, and often, say after cystoscopy, can go home the same day.

When a patient requiring operation has concomitant phthisis, diabetes, nephritis, or vomiting from intestinal obstruction, and reasons for preferring spinal anæsthesia are put to him, he usually consents readily.

*B.*—For those who dread not the operation but the (general) anæsthetic.

They are not a few, and for them spinal anæsthesia spells salvation. With them may be included a considerable percentage, mainly male, who while not afraid of narcosis are philosophical or phlegmatic enough to resign themselves to the numbing of their bodies. A few watch their operations in a mirror, inspect gall-stones or appendix at the time of their removal, and many marvel at the new "style."

*C.*—Those who wish to co-operate in what they are told is to their advantage but are imaginative and sensitive.

The anæsthetist needs to reassure and divert the patient's attention with appropriate conversation. A wet towel over the forehead and eyes, cotton wool in the ears and sips of water will afford comfort in the taciturn. The nostrils and mouth are better uncovered, and as cooling draughts relieve the occasional air hunger, there is no objection to opening doors and windows. Oxygen or carbon dioxide inhalations at some or most of the time are useful to both mind and body in some cases. I saw Dr. Falkner Hill at Manchester recently keeping a patient stuporous, though responsive to pinching the skin of her neck, throughout a partial gastrectomy. At the close he had only used one or two drachms of chloroform in addition to the spinal anæsthesia. Often it is not till the viscera are handled or when after ten to twenty minutes a sense of faintness may distress the patient as the circulatory balance alters that the need for additional anæsthesia arises. It may, however, in nervy types be better to give it earlier and get over the "struggling stage" that sometimes occurs before the peritoneal cavity is opened.

Pre-operative morphia and scopolamine were tried in the first hundred or two cases, but their routine use is of dubious advantage. In many cases the effect is negligible, in some it induces vomiting, and in all, since susceptibility decreases at the second dose, the potency of the drug is reduced when its effect is most appreciated by the patient, i.e. when the operation is over.

*D.*—Those who require oblivion regardless of drawbacks.

They are mainly in the first half of life. Such require gas or other general anæsthetic during the operations, and sometimes even before the thecal puncture. Thanks to the spinal anæsthesia they need only receive a trifling dose, and the surgeon does not depend for relaxation on the influence of what is inhaled. Obviously if these happen to be diabetic or jaundiced patients, or suffering from bronchitis or colds, they are at a disadvantage. Otherwise concomitant general anæsthesia is only a serious drawback, by complicating the picture somewhat if anxiety arises over lowered blood-pressure.

For this last reason I deem it necessary to proceed with caution in what at first seems the ideal solution of the anæsthetic problem in abdominal work, viz. premedication with basal narcotics like evipan, paraldehyde, or avertin, followed by spinal anæsthetic to gain perfect relaxation and operative tranquillity. Allured by such a combination I tried it on a succession of cases. The basal narcotic is apt to play tricks with patients. One nephrectomy patient needed several nurses to curb the violent and dangerous exertions which he exhibited for some twenty-four hours after operation, while another lady was found sitting on the floor voiding urine the night after appendicectomy.

However, such may be insufficient evidence on which to abandon such combinations. There are objections to giving (high) spinal anæsthesia to the narcotized. Correct placing for puncture of the theca is not always easy in the patient who while too drowsy to co-operate still resents the introduction of the needle. Secondly, the danger of basal narcosis in the susceptible patient is depression of the vital centres in the medulla. In the rare case where the resultant lowered vitality tends to be of a serious degree, an added fall of blood-pressure from the spinal anæsthetic makes it hard to revive the patient. Therefore where, for example, the paraldehyde-spinal combination is contemplated the possible slightly increased risk must be taken into account. Such a special indication as marked nervousness of the patient might well justify adoption of light inhalation anæsthesia, and I think the best routine adjuvant is nitrous oxide, which can be switched off to oxygen and carbon dioxide inhalations when danger threatens.

*E.*—A brief general anæsthetic prior to thecal puncture.

Ethyl chloride is eminently useful, and I have found evipan helpful. The patient thereafter is left to sleep, or tranquillized if necessary by occasional drops of anæsthetic during the operation. I have employed this with excellent results in infants and intractable children, where acute appendicitis has gone on to peritonitis, and where with such emergencies or, say, acute osteomyelitis colds or chest infections are also present. In lumbar sympathectomy, where relaxation is a great asset, I have found it helpful. A double advantage accrues from spinal anæsthesia in septic peritonitis. It saves adding to the existing toxæmia and often deflates and empties a loaded stagnant bowel. This facilitates subsequent saline absorption by this route, where the need for intestinal rest restricts fluids by mouth.

### THE PATIENT'S OPINION OF SPINAL ANÆSTHESIA.

What is the patient's opinion of spinal anæsthesia ? Once experienced, do patients elect to repeat it ? These are two vital questions on which I have collected some evidence from my wards at the Royal Infirmary during the months from April, 1935, to April, 1936.

| | Spinal Anæsthesia (alone). | Spinal with General Anæsthesia. | For. | Against. |
|---|---|---|---|---|
| Men .. | 90 | 20 | 95 | 15 |
| Women | 40 | 20 | 16 | 44 |

Obviously most of my male patients would prefer spinal if a second occasion arose. A few children have made its use a *sine qua non* for subsequent operations. I have repeated spinal anæsthesia the same day, and have used it again and again on some patients at their request—and without regret. A complaint of headache is quite a rarity, and no paralyses apart from a case of temporary squint and one of temporary deafness have appeared or are likely.

Thus in 1930 a woman aged 66, came with carcinoma of the left colon causing acute obstruction. For each of the three stages of her curative operation I gave spinocain. She still enjoys natural defæcation, good health, and does her usual work.

As a rule spinal anæsthesia is most favourable for patients in the latter part of life, which is fortunate, since both in their organs and the tissues involved in the operation they are less able to bear the added strain that general anæsthesia involves. They are calmer in mentality and start with the added advantage of a higher blood-pressure, although obviously they lack circulatory resilience. True, it is among these that the occasional anxieties and need for supra-renal injections arise. In no children or young folk has there been anxiety over lowered vitality.

It is a pleasant contrast to see "spinal" patients a few hours after their operation and compare their evident well-being with those who have been anæsthetized with ether and exhale it for a day or two after.

The help it affords in operating and the healing of wounds, often coupled with the appreciation of doctors and the gratitude of patients, encourage the continued use of spinal anæsthesia. Technique is satisfactory. Time is defining its scope.

SUMMARY.

1. A report is given on 1,529 cases of spinal anæsthesia. In approximately 90 per cent. no additional anæsthetic was used, and among these there were two deaths on the table. In the others some form of general or basal anæsthesia was added and one fatality occurred.

2. In a fraction per cent. it has been necessary to combat symptoms of shock by intravenous adrenalin, the effect of which is immediate and completely restorative.

3. The importance of a minimal dose is stressed, and use is made of varied dosage, level of injection and inclination of spine in controlling the distribution of anæsthesia. The phenomenon of *zonal* anæsthesia is described.

4. For the lumbo-sacral area, especially cystoscopic manipulation, it is unsurpassed; for the lower abdomen its advantages outweigh its disadvantages.

5. The debatable territory for its use is the upper abdomen. In this sphere it affords the greatest boon, where the patients are chronic invalids and ease of access is eagerly desired by the surgeon. Its chief danger, shock, which is here more apt to appear, is fully considered and its treatment described. Concomitant infiltration of the omentum and light general anæsthesia are often essential.

6. Contra-indications among acute and chronic cases are given.

7. In the acute emergency, where the surgeon is confronted with perforative peritonitis or intestinal obstruction often accompanied by some degree of an acute cold or bronchial infection, the avoidance of respiratory irritants is important, whilst the frequent evacuation and deflation of a stagnant colon under spinal anæsthesia leaves the way clear for adequate colonic feeding and alimentary rest.

8. Statistics are given showing that, while the majority of women do not, most men do prefer spinal anæsthesia.

9. The only sequela of note is headache. It has appeared in a mild form in a few per cent., and rarely has it been severe or lasted more than a few days.

10. Reference is made to the added responsibility incurred by the anæsthetist both in recognizing the indications for spinal or combined spinal and general anæsthesia and in piloting the patient to and through the operation.

### REFERENCES.

[1] Adams, A. Wilfrid, " Spinal Anæsthesia," *Brit. Med. Jour.*, 1931, i. 725–788.

[2] *Bri. Med. Jour.*, 1931, i. 869.

[3] Tidy, H. Letheby, " Treatment of Gastric and Duodenal Ulcer," *Brit. Med. Jour.*, 1936, i. 1,143–1,148.

[4] Shackle, J. W., " The Effect of Ether Anæsthesia on the Blood Urea," *Journal of Clinical Research*, 1932, xviii. 138 *et seq.*

